# Automated cell tracking using StarDist and TrackMate

**DOI:** 10.12688/f1000research.27019.1

**Published:** 2020-10-28

**Authors:** Elnaz Fazeli, Nathan H. Roy, Gautier Follain, Romain F. Laine, Lucas von Chamier, Pekka E. Hänninen, John E. Eriksson, Jean-Yves Tinevez, Guillaume Jacquemet

**Affiliations:** 1 Laboratory of Biophysics, Institute of Biomedicine, Faculty of Medicine, University of Turku, Turku, Finland; 2 Department of Pathology and Laboratory Medicine, Children's Hospital of Philadelphia Research Institute, Philadelphia, PA 19104, USA; 3 Turku Bioscience Centre, University of Turku and Åbo Akademi University, Turku, Finland; 4 Cell Biology, Faculty of Science and Engineering, Åbo Akademi University, Turku, Finland; 5 MRC-Laboratory for Molecular Cell Biology, University College London, London, UK; 6 The Francis Crick Institute, London, UK; 7 Image Analysis Hub, Institut Pasteur, Paris, France

**Keywords:** Cell migration, Image analysis, StarDist, TrackMate, Deep-learning, Automated tracking

## Abstract

The ability of cells to migrate is a fundamental physiological process involved in embryonic development, tissue homeostasis, immune surveillance, and wound healing. Therefore, the mechanisms governing cellular locomotion have been under intense scrutiny over the last 50 years. One of the main tools of this scrutiny is live-cell quantitative imaging, where researchers image cells over time to study their migration and quantitatively analyze their dynamics by tracking them using the recorded images. Despite the availability of computational tools, manual tracking remains widely used among researchers due to the difficulty setting up robust automated cell tracking and large-scale analysis. Here we provide a detailed analysis pipeline illustrating how the deep learning network StarDist can be combined with the popular tracking software TrackMate to perform 2D automated cell tracking and provide fully quantitative readouts. Our proposed protocol is compatible with both fluorescent and widefield images. It only requires freely available and open-source software (ZeroCostDL4Mic and Fiji), and does not require any coding knowledge from the users, making it a versatile and powerful tool for the field. We demonstrate this pipeline's usability by automatically tracking cancer cells and T cells using fluorescent and brightfield images. Importantly, we provide, as supplementary information, a detailed step-by-step protocol to allow researchers to implement it with their images.

## Introduction

**Figure 1.**
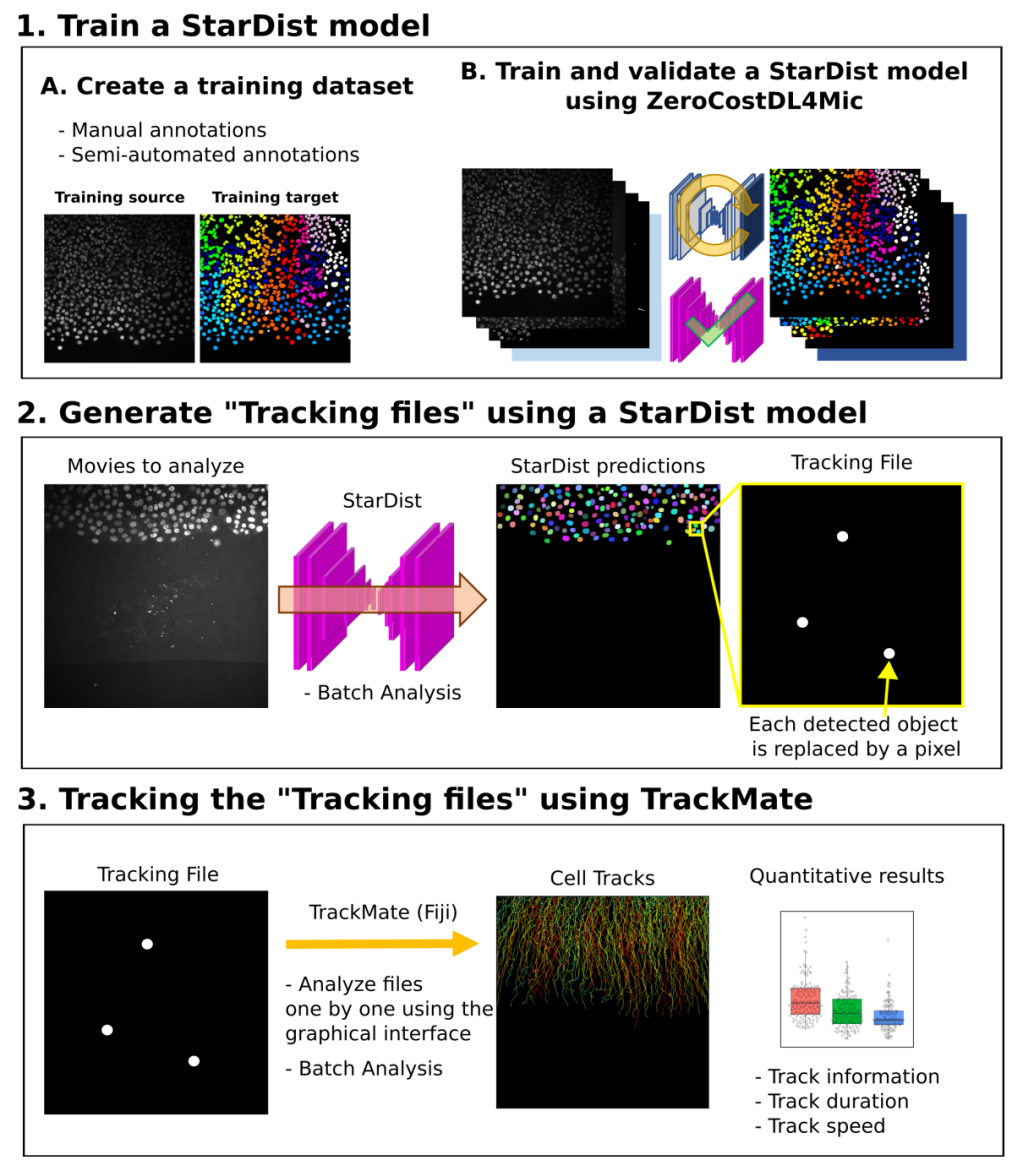
Workflow depicting how StarDist and TrackMate can be combined to track cells automatically.

The study of cell motility typically involves recording cell behavior, using live-cell imaging, and tracking their movement over time ^1, 2^. To enable the analysis of such data, various software solutions have been developed ^3– 9^. However, despite the availability of these computational tools, manual tracking remains widely used among researchers due to the difficulty in setting up fully automated cell tracking analysis pipelines. Automated tracking pipelines share a typical workflow that starts with a segmentation strategy that identifies the objects to track in each image. Tracking algorithms are then used to link these objects between frames. One challenging aspect of an automated tracking pipeline is often achieving an accurate segmentation of the objects to track. One option to facilitate cell segmentation is to label their nuclei, using fluorescent dyes or protein markers. Nuclei can then be automatically segmented using intensity-based thresholding. However, this approach tends to become inaccurate when images are noisy or when the cells to track are very crowded ^10^. Deep-Learning approaches have demonstrated their robustness against these two issues ^11^. In this work, we present a new analysis workflow that builds upon a Deep-Learning segmentation tool and a cell tracking tool to achieve robust cell tracking in cell migration assays. We combine StarDist, a powerful deep learning-based segmentation tool, and TrackMate, a user-friendly tracking tool, into a tracking pipeline that can be used without requiring expertise in or specialized hardware for computing ( Figure 1) ^12– 15^.

## Methods

### Pipeline

The use of deep learning networks, such as StarDist, often requires the user to train or retrain a model using their images. While high-quality StarDist pre-trained models are readily available, they are likely to underperform when used on different data with, e.g., different staining, noise, and microscope type ^15^. To train StarDist models, we took advantage of the ZeroCostDL4Mic platform, allowing researchers to train (and retrain), validate, and use deep learning networks ^15^. Importantly, the ZeroCostDL4Mic StarDist 2D notebook can directly output a file containing all the nuclei's geometric center coordinates (named tracking files), that can be used as input for TrackMate ( Figure 1). Therefore, our proposed pipeline can be divided into three parts ( Figure 1; *Extended data* ^16^). 1) First, a StarDist model is trained using the ZeroCostDL4Mic platform. This part needs to be performed only once for each type of data. 2) Second, the trained StarDist model is used to segment the object to track and generate Tracking files. 3) Finally, the tracking files can be used in TrackMate to track the identified objects.

**Figure 2.**
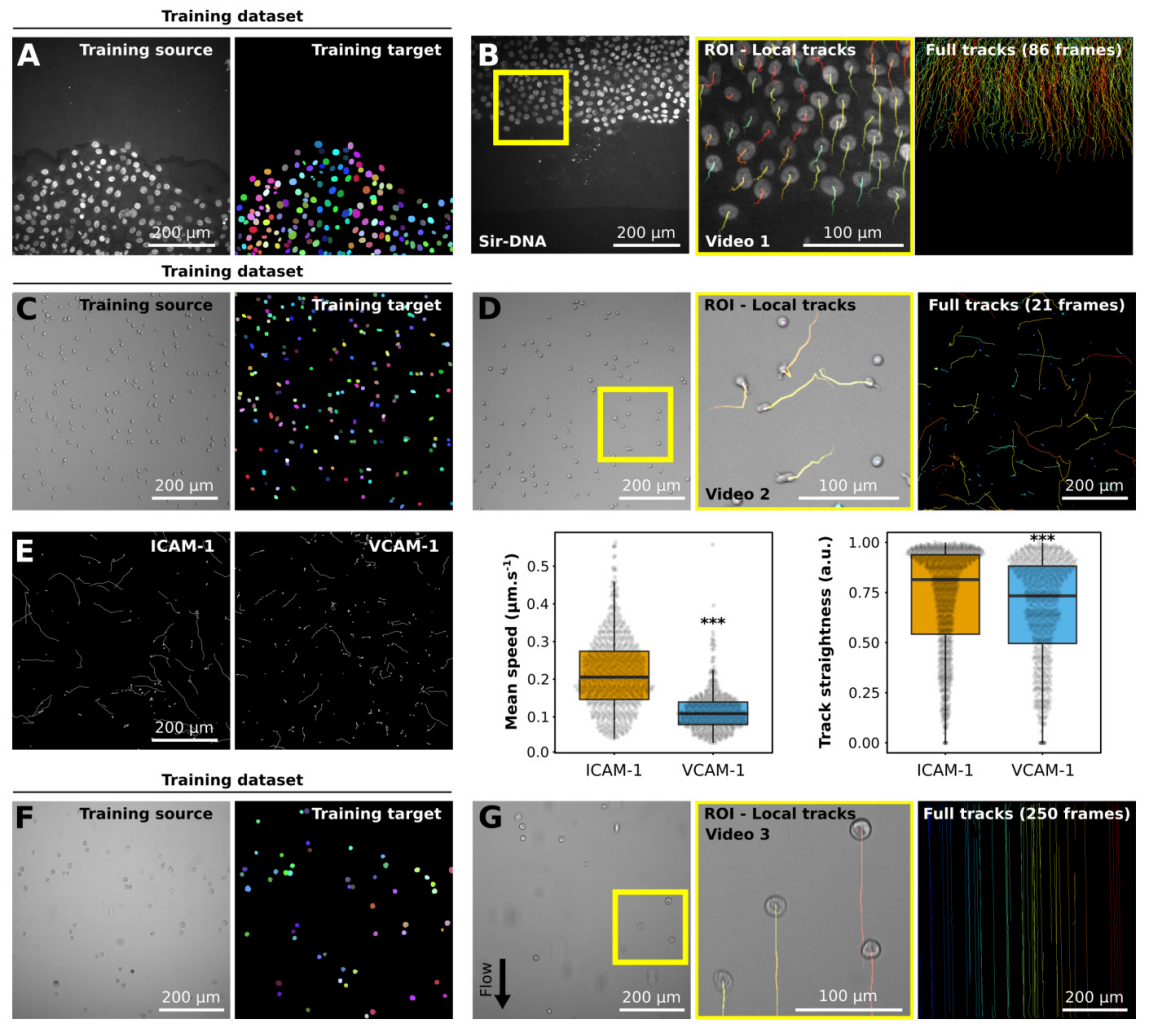
Example of datasets analyzed using StarDist and TrackMate. ( **A**, **B**) Migration of MCF10DCIS.com, labeled with Sir-DNA, recorded using a spinning disk confocal microscope and automatically tracked. Examples of images used to train StarDist ( **A**), and an example of results obtained using automated tracking are displayed ( **B**, Video 1). The yellow square indicates a magnified ROI, where the local track of each nucleus is displayed. The full cell tracks are displayed on the left. Tracks are color-coded as a function of their maximum instantaneous velocity (blue slow, red fast tracks). ( **C**– **E**) Migration of activated T cell plated on VCAM-1 or ICAM-1, recorded using a brightfield microscope and automatically tracked. Examples of images used to train StarDist ( **C**) and an example of results obtained using automated tracking are displayed ( **D**, Video 2). ( **E**) Comparison of the migration of activated T cells on VCAM-1 or ICAM-1. Track mean speed and track straightness were quantified. Data are displayed as boxplots. *** p-value = <0.001, p-values were determined using a randomization test. ( **F**, **G**) Cancer cells flowing in a microfluidic chamber, recorded live using a brightfield microscope and automatically tracked (Video 3). Examples of images used to train StarDist ( **F**), and an example of results obtained using automated tracking are displayed ( **G**). The full tracks shown here were color-coded as a function of their x coordinate.

Training a StarDist model requires a set of images and their corresponding masks ( Figure 1 and Figure 2). Generating a training dataset is by far the most time-consuming part of the analysis pipeline presented here as it requires the manual annotations of the images to analyze ( *Extended data:* Supplementary protocol ^16^). For instance, to generate the training datasets presented in Figure 2, each cell/nuclei contour was drawn manually using the freehands selection tool in Fiji. The creation of a high-quality training dataset is a critical part of the process as it will impact the specificity and performance of the StarDist model. However, the generation of a training dataset is only required once per dataset type. If a StarDist model already exists for similar images it can be used to significantly accelerate the creation of the training dataset via semi-automated annotation (see *Extended data:* Supplementary protocol ^16^).

One of our analysis pipeline's key features is that, once a StarDist model has been satisfactorily trained, movies of migrating cells can efficiently be processed in batch. Indeed, while individual tracking files can be analyzed one by one using the TrackMate graphical interface, we also provide a Fiji macro to analyze a folder containing multiple tracking files. Our batch processing macro will provide basic quantitative information for each track, including median and maximal speeds. If more information is needed, the tracking results generated by our script are directly compatible with the Motility lab website, where they can be further processed ^17^.

### Implementation and operation

The described image analysis pipeline is composed of a Jupiter notebook optimized to run in Google Colab (ZeroCostDL4Mic framework ^15^) and a Python script that can run in Fiji ^14^. A step-by-step protocol describing how to use our analysis pipeline is provided as *Extended data* ^16^.

## Use case

To illustrate our analysis pipeline's functionality and flexibility, we first trained a StarDist model to analyze the behavior of breast cancer cells migrating collectively ( Figure 2A; *Extended data:* Video 1 ^16^). The cancer cell's nuclei were fluorescently labeled, and the cells imaged using fluorescence-based microscopy. The creation of the training dataset used in this example was greatly facilitated by the availability of a StarDist model, released by the StarDist creators, capable of segmenting fluorescent nuclei. In this case, the StarDist Fiji plugin was used to segment the location of nuclei in the training images, and all miss-annotations were manually corrected ( *Extended data:* Supplementary protocol ^16^).

Video 1: Automated tracking of breast cancer cell migrating collectively **Video 1: Automated tracking of breast cancer cell migrating collectively.** MCF10DCIS.com cells, labeled with Sir-DNA, were recorded using a spinning disk confocal microscope and automatically tracked using StarDist and TrackMate. Local tracks are displayed. Click here for additional data file. Copyright: © 2020 Fazeli E et al. 2020

To highlight that our pipeline can also be used to analyze brightfield images, we generated a StarDist model to track T cells migrating on ICAM-1 or VCAM-1 ( Figure 2C–E; *Extended data:* Video 2 ^16^). Importantly, automated analysis of these data could reproduce the results obtained via manual tracking ^19^.

Video 2: Automated tracking of T cell migrating on ICAM-1 Video 2: Automated tracking of T cell migrating on ICAM-1. Activated T cell plated ICAM-1 were recorded using a brightfield microscope and automatically tracked using StarDist and TrackMate. Local tracks are displayed. Click here for additional data file. Copyright: © 2020 Fazeli E et al. 2020

Finally, we used our pipeline to automatically track non-adherent cancer cells flowing in a microfluidic chamber ( Figure 2F and G; *Extended data:* Video 3 ^16^). In this case, automated tracking is especially useful due to the very high number of frames to analyze. For the last two examples, no suitable pre-trained StarDist models were available. Therefore, to generate the training datasets, we manually annotated 20 images and trained a first StarDist model. This model was then used to accelerate the annotation of the rest of the training images.

Video 3: Automated tracking of cancer cells flowing in a microfluidic chamber **Video 3: Automated tracking of cancer cells flowing in a microfluidic chamber.** AsPC1 pancreatic cancer cells flowing in a microfluidic chamber were recorded live using a brightfield microscope and automatically tracked using StarDist and TrackMate. Local tracks are displayed. Click here for additional data file. Copyright: © 2020 Fazeli E et al. 2020

### Use case dataset creation

***Breast cancer cell dataset*.** MCF10DCIS.com cells were described previously ^15, 22^. DCIS.COM lifeact-RFP cells were incubated for 2h with 0.5 µM SiR-DNA (SiR-Hoechst, Tetu-bio, Cat Number: SC007) before being imaged live for 14 h using a spinning-disk confocal microscope (1 picture every 10 min). The spinning-disk confocal microscope used was a Marianas spinning disk imaging system with a Yokogawa CSU-W1 scanning unit on an inverted Zeiss Axio Observer Z1 microscope (Intelligent Imaging Innovations, Inc.) equipped with a 20x (NA 0.8) air, Plan Apochromat objective (Zeiss).

***T cell dataset*.** Lab-Tek 8 chamber slides (ThermoFisher) were coated with 2 μg/mL ICAM-1 or VCAM-1 overnight at 4°C ^19^. Activated primary mouse CD4+ T cells were washed and resuspended in L-15 media containing 2 mg/mL D-glucose. T cells were then added to the chambers, incubated 20 min, gently washed to remove all unbound cells, and imaged. Imaging was done using a 10x phase contrast objective at 37°C on a Zeiss Axiovert 200M microscope equipped with an automated X-Y stage and a Roper EMCCD camera. Time-lapse images were collected every 30 sec for 10 min using SlideBook 6 software (Intelligent Imaging Innovations).

***Flow chamber dataset*.** Cancer cells (500,000 cells/ml in PBS) were perfused at a speed of 300 µm/sec using a peristaltic pump (ISMATEC MS12/4 analogic) and a homemade tubing system (Ismatek 3-Stop tubes and Ibidi ^®^ tubings and connectors) in a microchannel (Ibidi ^®^ µ-slides400 LUER). Images were acquired with a brightfield microscope (Zeiss Laser-TIRF 3 Imaging System, Carl Zeiss) and a 10X objective.

### Data display and statistical analyses

Box plots were generated using PlotsOfData ^23^. Randomization tests were performed using the online tool PlotsOfDifferences ^24^.

## Conclusions

Here we show that StarDist and TrackMate can be integrated seamlessly and robustly to automate cell tracking in fluorescence and brightfield images. We envision that this pipeline can also be applied to any circular or oval-shaped objects. However, we acknowledge that using brightfield images may not always work directly with our pipeline, especially if cells display complex and interchanging shapes, since StarDist is mostly designed to detect round or compact shapes. In this case, other tools, such as Usiigaci, could also be considered ^8^. Still, brightfield images could also be artificially labeled using deep learning, transforming the brightfield dataset into a pseudo-fluorescence one, as can be done with ZeroCostDL4Mic already ^15^. The pipeline described here is currently limited to the tracking of objects in 2D. However, a similar workflow can be applied to 3D datasets as both StarDist and TrackMate can accommodate 3D images ^12, 13, 25^.

## Data availability

### Underlying data

Zenodo: Combining StarDist and TrackMate example 1 - Breast cancer cell dataset, http://doi.org/10.5281/zenodo.4034976 ^26^

Zenodo: Combining StarDist and TrackMate example 2 - T cell dataset, http://doi.org/10.5281/zenodo.4034929 ^27^

Zenodo: Combining StarDist and TrackMate example 3 - Flow chamber dataset, http://doi.org/10.5281/zenodo.4034939 ^28^

### Extended data

Zenodo: Combining StarDist and TrackMate - Extended data, http://doi.org/10.5281/zenodo.4091467 ^16^.

This project contains the following extended data:

- Supplementary protocol
- **Video 1: Automated tracking of breast cancer cell migrating collectively.** MCF10DCIS.com cells, labeled with Sir-DNA, were recorded using a spinning disk confocal microscope and automatically tracked using StarDist and TrackMate. Local tracks are displayed.
- **Video 2: Automated tracking of T cell migrating on ICAM-1.** Activated T cell plated ICAM-1 were recorded using a brightfield microscope and automatically tracked using StarDist and TrackMate. Local tracks are displayed.
- **Video 3: Automated tracking of cancer cells flowing in a microfluidic chamber.** AsPC1 pancreatic cancer cells flowing in a microfluidic chamber were recorded live using a brightfield microscope and automatically tracked using StarDist and TrackMate. Local tracks are displayed.

Data are available under the terms of the Creative Commons Attribution 4.0 International license (CC-BY 4.0).

## Software availability

Source code available from: https://github.com/HenriquesLab/ZeroCostDL4Mic

Archived source code at time of publication: http://doi.org/10.5281/zenodo.4091474 ^26^

License: MIT license.
