# Supplementary material for: Automated cell tracking using StarDist and TrackMate
Source: F1000Res. 2020 Oct 28;9:1279. [Version 1] doi: 10.12688/f1000research.27019.1 (PMC7670479; doi:10.12688/f1000research.27019.1)
Supplement: Peer review history

# Peer review: Reviewer response for version 1

**DOI:** 10.5256/f1000research.29845.r73862

**Published:** 2020-11-16

**Authors:** Vinay Swaminathan

**Affiliations:** 1 Lund University, Lund, Sweden

In this article, Fazeli *et al. *combine deep-learning segmentation tools with open access cell tracking platforms to show the feasibility for automated cell tracking and large population motility analysis. Using this approach, the authors here clearly describe and validate this process in fluorescent movies of collective cell migration as well as single cell bright-field cell migration datasets. While the description of methods is very clear (and special kudos to the authors for transparency and availability), and its applicability very apparent, a few other potential points to consider are as follows:

1. A comparison on a given dataset between trackmate with StarDist based segmentation versus canonical segmentation methods of the nuclei. How much better does it perform? This could be in terms of accuracy/precision or time taken to analyze. 
2. Related to point 1: The power of StarDist based nuclear segmentation is its ability in crowded environments as well as where there is poor signal in the fluorescent channels. Is there a way to explicitly show that when there is poor signal or bleaching during a movie, StarDist combined with trackmate does a better job?
3. One of the hardest objects to segment are bright-field objects which is why the field has been relying on hand tracking of cell migration movies taken with bright-field. I think the authors should underscore this and highlight that this method is overcoming this big challenge enabling larger population analysis.

Are the conclusions about the tool and its performance adequately supported by the findings presented in the article?

Yes

Is the rationale for developing the new software tool clearly explained?

Yes

Is the description of the software tool technically sound?

Yes

Are sufficient details of the code, methods and analysis (if applicable) provided to allow replication of the software development and its use by others?

Yes

Is sufficient information provided to allow interpretation of the expected output datasets and any results generated using the tool?

Yes

Reviewer Expertise:

Cell migration, mechanobiology, Cancer.

I confirm that I have read this submission and believe that I have an appropriate level of expertise to confirm that it is of an acceptable scientific standard.

# Peer review: Reviewer response for version 1

**DOI:** 10.5256/f1000research.29845.r73865

**Published:** 2020-11-11

**Authors:** Ignacio Arganda-Carreras

**Affiliations:** 1 Department of Computer Science and Artificial Intelligence, Basque Country University, San Sebastian, Spain

The authors propose the combination of open-source tools (namely StarDist and TrackMate) for the automatic tracking of cells in fluorescence and brightfield images in 2D. Moreover, they provide a step-by-step workflow to process videos in a batch mode using exclusively free tools. They evaluate its performance by comparing the results obtained using such a workflow with those of manual tracking from an already published public dataset.

 The paper is very well written, concise and straight to the point, with a special emphasis on reproducibility.

 As pointed out in the conclusions, one of the limitations of the proposed approach is inherent to the type of objects that StarDist can properly segment (basically round). However, that doesn't prevent this pipeline from being extremely useful for a broad spectrum of cell tracking problems. Moreover, the adaptation of the pipeline to 3D images seems pretty straightforward.

 Something interesting that is not mentioned in the paper is how well the workflow would perform in the presence of cell divisions of apoptosis. That could be easily tested using some of the datasets from the Cell Tracking Challenge (http://celltrackingchallenge.net/2d-datasets/).

 Minor comments:

- Please homogenize how you write "deep learning", which appear sometimes as "Deep-Learning" and sometimes as "deep learning".

Are the conclusions about the tool and its performance adequately supported by the findings presented in the article?

Yes

Is the rationale for developing the new software tool clearly explained?

Yes

Is the description of the software tool technically sound?

Yes

Are sufficient details of the code, methods and analysis (if applicable) provided to allow replication of the software development and its use by others?

Yes

Is sufficient information provided to allow interpretation of the expected output datasets and any results generated using the tool?

Yes

Reviewer Expertise:

Computer Vision, Bioimage Analysis.

I confirm that I have read this submission and believe that I have an appropriate level of expertise to confirm that it is of an acceptable scientific standard, however I have significant reservations, as outlined above.

# Peer review: Reviewer response for version 1

**DOI:** 10.5256/f1000research.29845.r73864

**Published:** 2020-11-11

**Authors:** Lachlan W. Whitehead

**Affiliations:** 1 The Walter and Eliza Hall Institute of Medical Research, Parkville, Vic, Australia

This article presents a pipeline for analyzing cell migration in a variety of contexts by combining several complimentary techniques. Utilising stardist for cell detection (provided cells are round/have nuclei staining), and trackmate for connecting the detected nuclei over time - a start-to-finish protocol is described allowing a microscopist with little image analysis knowledge to be able to quantify their experiment. 

 The authors do an admirable job of describing the required steps of the analysis pipeline, including an introduction to Jupyter Notebooks and the ZeroCostDL4Mic workflows to train a custom Stardist model. They also provide a FIJI macro for batch analysis, potentially saving a researcher many hours of human analysis time. 

 Indeed, as all of these methods are published and validated already my only (small) criticism has to do with the training of the stardist models. As the authors rightly note, this is the most time consuming part of the analysis and as a result I would have liked to see some mention of how much manual time is required. For instance, while the article suggests training a small 20 image dataset and then using transfer learning to speed up the remaining annotation, the number of remaining images is not described. While the amount of training required is likely to vary across experiments, I think readers would benefit from knowing when considering this pipeline how many cells (rather than image fields of view) they are likely to be required to manually annotate. Perhaps for each dataset where a model was trained the authors could specify the size of the training dataset (in both images and number of cells). 

 Overall, this is a clear description of several powerful tools being combined into a very useful and versatile workflow.

Are the conclusions about the tool and its performance adequately supported by the findings presented in the article?

Yes

Is the rationale for developing the new software tool clearly explained?

Yes

Is the description of the software tool technically sound?

Yes

Are sufficient details of the code, methods and analysis (if applicable) provided to allow replication of the software development and its use by others?

Yes

Is sufficient information provided to allow interpretation of the expected output datasets and any results generated using the tool?

Yes

Reviewer Expertise:

bioimage analysis

I confirm that I have read this submission and believe that I have an appropriate level of expertise to confirm that it is of an acceptable scientific standard.
